# Nighttime ionospheric irregularity during intense geomagnetic storm events over the Europe-African longitudinal sector

**DOI:** 10.1016/j.heliyon.2024.e38138

**Published:** 2024-09-23

**Authors:** Yibekal Kassa, Ambelu Tebabal, Baylie Damtie

**Affiliations:** a College of Science, Department of Physics, Washera Geospace and Radar Science Research Laboratory (WaGRL), Bahir Dar University, Ethiopia; b College of Natural and Computational Science, Department of Physics, Debre Markos University, Ethiopia; c Institute of Geophysics, Space Science and Astronomy, Addis Ababa University, Ethiopia

**Keywords:** Geomagnetic storm, Longitudinal sectors, Irregularity, ROTI, RODI

## Abstract

Radio communication and navigation systems can be severely impacted by irregularities in the ionosphere. There is still much to learn about how geomagnetic storms affect the occurrence of these irregularities. Ionosphere studies in different regions, particularly the equatorial and low-latitudes, are necessary to enhance the forecasting of this phenomenon. This study investigates the effect of intense geomagnetic storm events of August 27, October 7 and December 22, 2015, on nighttime ionospheric irregularities. Data collected from the receivers of the Global Navigation Satellite Systems (GNSS) in specific longitudinal sector of 15^∘^W – 0^∘^, 0^∘^ – 15^∘^E, 15^∘^E – 30^∘^E and 30^∘^E – 45^∘^E, as well as from the Swarm constellations in the longitude range of $30^{\circ }$W – $70^{\circ }$E and the latitude range of $50^{\circ }$S – $50^{\circ }$N, have been used. The rate of change of the total electron content (TEC) index (ROTI) and the rate of change of the electron density index (RODI) were analyzed. In the main phases of the storms, at equatorial and low-latitude regions we observed ionospheric irregularities in the African longitudinal sectors of 15^∘^W – 0^∘^, 0^∘^ – 15^∘^E and 30^∘^E – 45^∘^E in the three storm events. The observed ionospheric irregularities were more pronounced in the western than eastern regions. These irregularities were possibly driven by the Prompt Penetration Electric Fields (PPEFs) that point east to west during nighttime. Ionospheric irregularities were inhibited in the selected storm periods over the middle latitudes in the African sector. In the longitudinal sector of 15^∘^E – 30^∘^E, we obtained inhibition of irregularity in the study periods. In the case of the top-side ionosphere, we observed enhanced and depleted electron density during the storm in the low latitude and equatorial regions. In the low latitude and equatorial regions, there were electron density fluctuations within the range of $15^{\circ }$N and $15^{\circ }$S, indicating significant top-side irregularities.

## 1 Introduction

Intense geomagnetic storms lead to ionospheric irregularities [1], as space weather conditions highly influence Earth's ionosphere. Ionospheric irregularities refer to small structures within the ionospheric plasma density that are typically orientated in a way that causes variations in rapid changes in plasma density are expected throughout the geomagnetic field, but they will happen slowly or not at all along [2]. Ionospheric disturbances vary from storm to storm as a result of the intricate interaction that takes place during geomagnetic storms between the magnetosphere, ionosphere, and thermosphere. During this period, significant perturbations occur at equatorial as well as higher latitudes [3]. In addition, disturbances manifest more prominently and frequently in the nighttime sector of the Earth where high-latitude energy injection occurs and the neutral winds favor an equatorward direction [4], [5].

Ionospheric irregularities primarily occur at night in low-latitude and equatorial regions [6], [7], [8]. In these regions, ionospheric plasma density anomalies, or Equatorial plasma bubbles (EPBs), are thought to be mainly caused by Rayleigh-Taylor instabilities (RT-instability) [9], [10], [11]. These irregularities range in scale from a few centimeters to thousands of kilometers [12], [13]. The mid-latitude ionosphere, on the other hand, is less irregular due to the lack of RT-instability typical of equatorial latitudes and the lack of direct connection to the strong forcing from the magnetosphere, as observed at auroral latitudes [14], [15]. So, space weather events have an impact on the ionosphere at middle latitudes both directly and through couplings with polar and equator regions [16], [17]. Small-scale ionospheric irregularities can modulate the propagation of radio waves, significantly impacting communications, navigation, and radar systems [18], [19], [20], [21], [22]. Therefore, gaining a better understanding of the dynamics of the ionosphere during geomagnetic storms is crucial to enhancing our knowledge of how susceptible technology is to space weather.

Many studies have explored ionospheric irregularities and scintillation properties through ground-based observations across diverse longitudinal sectors and solar-geomagnetic periods (e.g., [23], [24], [25]). Seba and Gogie [26] analyzed GPS-SCINDA data from August 2010 to July 2011 at Bahir Dar station, Ethiopia, showing post-sunset scintillation peaks, notably during the March equinox. Dugassa et al. [27] investigated irregularities in equatorial/low-latitude regions during 2012 – 2013, noting significant variations during geomagnetic storms. Olwendo et al. [15] examined monthly irregularity variations during solar cycle 24's declining phase across the African low-latitude region, identifying strong patterns, particularly in the western region. Paznukhov et al. [24] longitudinally studied Equatorial Plasma Bubbles (EPBs) and GPS scintillations over equatorial Africa in 2010, finding a correlation between scintillation strength and plasma bubble presence. To date, due to increasing interest in space weather applications, numerous low Earth orbit (LEO) satellites, including the SWARM mission, have been launched to detect topside ionosphere parameters, aiding in our understanding of its physical state. In a recent study, [28] investigated the mapping of turbulence indices in the topside ionosphere, while [17] examined middle latitude on September 8, 2017, during a magnetic storm, there were plasma bubbles over China and other regions. Cherniak and Zakharenkova [29] studied how the geomagnetic storm that occurred on August 25 – 26, 2018 caused ionospheric irregularities across middle and equatorial latitudes. However, the research mentioned above, along with others not cited, did not account for geographical (longitudinal sectors at various latitudes) and temporal variations during nighttime geomagnetic storm events. Moreover, these investigations were limited to a small number of observational sites and did not utilize more than two GPS stations. Integrating ionospheric parameters observed by both GNSS ground-based stations and LEO satellites has the potential to significantly enhance our understanding of the structure of the ionosphere.

In present study, we aim to characterize nighttime ionospheric irregularities in the initial, main, and recovery phases of intense storm events occurring on August 27, October 7, and December 20, 2015. While prior research has shed light on ionospheric irregularities during geomagnetic storms, nighttime conditions have not been thoroughly examined. Our focus is to demonstrate the variations in nighttime ionospheric irregularities across different latitudes within longitudinal sectors ranging from $15^{\circ }$W to $0^{\circ }$, $0^{\circ }$ to $15^{\circ }$E, $15^{\circ }$E to $30^{\circ }$E, and $30^{\circ }$E to $45^{\circ }$E using multiple ground-based GPS-TEC and LEO satellite observations.

## 2 Data and methods

**Figure 1:**
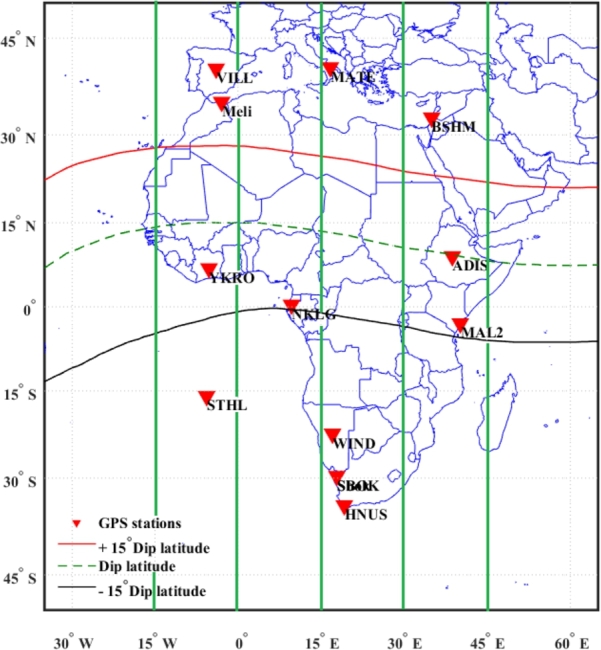
Distribution of the GPS receivers in the study area.

Within the present study, we examined the TEC data from twelve GPS receivers situated over the middle, equatorial, and low-latitude regions of Europe-Africa longitudinal sector in the year 2015 for the selected storm periods of August 25 – 29, October 5 – 9, and December 18 – 22. Gonzalez et al. [30] classification of geomagnetic storms into weak, moderate, and intense categories was based on the distribution of the hourly values that were observed. A storm is classified as weak if its minimum Dst value falls between 30 and 50 nT, which accounts for 25% of the hourly distribution of the observed values; moderate storms are those that fall between 50 and 100 nT, which accounts for 8% of the values; and intense storms are those that fall below 100 nT, which accounts for 1% of the observed values. Drawing from this categorization, we have chosen for our case study the August 27, October 7, and December 20 geomagnetic storm events, which are listed in Table 1. The locations of the GPS stations used in this investigation are displayed in Fig. 1, and their geographic and geomagnetic coordinates are listed in Table 2. The data is provided by the University of NAVSTAR Consortium (UNAVCO) in RINEX (Receiver Independent Exchange format: http://www.unavco.org). Slant Total Electron Content (STEC) is estimated from GPS transmissions using the method developed by [31]. STEC is converted to vertical TEC using the shell mapping model [32]. The temporal resolution of the TEC time series is 30 s. The signals with elevation angle < $30^{\circ }$ are excluded from the analysis to eliminate the possible tropospheric and multi-path effects [9].

**Table 1:** Characteristics of the geomagnetic storms used in this study.

Month of the year 2015	Storm Days	Dst index (nT)	IMF-Bz (nT)
August	25 – 29	- 103	- 11.5
October	5 – 9	- 130	- 11.3
December	18 – 22	- 166	- 18.7

**Table 2:** Geographic and geomagnetic Coordinates of the GPS stations.

Longitudinal sector	Station Name/Country	Station code	Geographic Latitude	Geographic Longitude	Geomagnetic Latitude	Geomagnetic Longitude
	Yamoussoukro, Cote d'Ivoire	YKRO	6.87^∘^N	5.24^∘^W	2.86^∘^S	77.22^∘^E
15^∘^W – 0^∘^	Villafranca	VILL	40.44^∘^N	3.95^∘^W	33.27^∘^N	79.16^∘^E
	Melilla	MELI	35.28^∘^N	2.95^∘^W	25.64^∘^N	77.43^∘^E
	St.Helena	STHL	15.94^∘^S	5.66^∘^W	25.15^∘^S	74.19^∘^E

0^∘^ – 15^∘^E	N'Koltang(Gabon)	NKLG	0.35^∘^N	9.67^∘^E	8.04^∘^S	81.05^∘^E

	Matera	MATE	40.64^∘^N	16.70^∘^E	33.99^∘^N	90.17^∘^E
15^∘^E – 30^∘^E	Windhoek	WIND	22.57^∘^S	17.08^∘^E	33.15^∘^S	84.63^∘^E
	Springbok	SBOK	29.66^∘^S	17.87^∘^E	39.02^∘^S	82.84^∘^E
	Hermanus	HNUS	34.42^∘^S	19.22^∘^E	42.34^∘^S	82.14^∘^E

	Binyamin Shmuter Memorial site	BSHM	32.77^∘^N	35.02^∘^E	25.99^∘^N	106.61^∘^E
30^∘^E – 45^∘^E	Addis Ababa, University	ADIS	9.03^∘^N	38.76^∘^E	0.16^∘^N	110.45^∘^E
	Malindi, Kenya	MAL2	2.99^∘^S	40.19^∘^E	12.41^∘^S	111.85^∘^E

Ionospheric irregularities have been examined using the rate of change of the TEC index (ROTI) [33]. As stated in [34], ROTI is a parameter that is determined by the rate of change of TEC (ROT) that is derived from the TEC time variation. These indices can be utilized as a useful proxy to describe different aspects of the ionospheric irregularity [35].

A parameter ROT, as defined by [34], is the rate of change of TEC obtained from the time variation of TEC and estimated by

(1) $$ROT(t)=\frac{TEC(t+\delta t)-TEC(t)}{\delta t},$$

where *t* is the time of epoch. In Eq. (1), *TEC* is in TECU and *δt* is the time difference between the epoch = 30 s, hence, ROT is converted to TECU/min multiplying it by 60 s.

According to [34], the ROTI index is the standard deviation of ROT over a specific time interval. It was computed by taking the running window of size 5-minutes and given by

(2) $$ROTI(t_{k})=\frac{1}{N-1}\sum_{t_{k}=t-\frac{\delta t}{2}}^{t_{k}=t+\frac{\delta t}{2}}\sqrt{|ROT(t_{k})-\langle ROT\rangle {|}^{2}},$$

where *N* is the number of visible satellites, 〈ROT〉 is the average of TEC over a specific time interval and ROT is given by Eq. (1).

Using ROTI values, we categorize the degree of ionospheric irregularities as follows: No irregularity is seen when ROTI is less than 0.25 TECU/min; weak irregularity is shown when ROTI is between 0.25 and 0.5 TECU/min; moderate irregularity is observed when ROTI is between 0.5 and 1 TECU/min; and strong irregularity is when ROTI is greater than 1 [36], [37].

We have also used data from Swarm three satellites with different orbital altitudes [38] for studying irregularities in the topside ionosphere from VirES for Swarm (https://vires.services). The Swarm constellation consists of three identical near-polar satellites, namely A, B, and C. While Swarm B orbits the Earth approximately 50 km higher than Swarm A and C, Swarm A and C fly side by side at an altitude of around 450 km with a longitudinal spacing of $1.4^{\circ }$ (approximately 150 km). The three satellites, A, C, and B, take around 133 days, 141 days, and 141 days, respectively, to complete a full day of Local Time (LT). In this investigation, the onboard Langmuir Probe, with a sampling rate of 2 Hz, was used to provide in-situ electron density (*Ne*) measurements.

Data obtained from Swarm satellites has been utilized to detect plasma bubbles, known as the Ionospheric Bubble Index (IBI). These irregularities in the ionosphere are most commonly observed after sunset in regions with low latitudes [39]. To evaluate the extent of these irregularities, the Rate of Change of Electron Density (ROD) is calculated by determining the time derivatives of electron density along the satellite's orbital path.

The Rate of change of electron density (ROD) is defined by

(3) $$ROD(t)=\frac{Ne(t+\delta t)-Ne(t)}{\delta t}$$

where the electron density at time *t* is represented by Ne(t). Only time consecutive data are used to generate ROD values. When we consider the 2 Hz sampling rate of the Swarm satellites, *δt* becomes 0.5 s.

Rate of change of Electron Density Index (RODI), which can be estimated from in-situ electron density measurements, is the ionospheric index for identifying and assessing the irregularities of the topside ionosphere. RODI index has been used by many authors to quantify the level of irregularities in the topside ionosphere [34], [40], [41], [42], [43], [44].

RODI index is defined in a similar way to ROTI by substituting ROD in the place of ROT [42]. It is the standard deviation of ROD and can be estimated in a running window of 10 s given by

(4) $$RODI(t_{k})=\frac{1}{N-1}\sum_{t_{k}=t-\frac{\delta t}{2}}^{t_{k}=t+\frac{\delta t}{2}}\sqrt{|ROD(t_{k})-\langle ROD\rangle {|}^{2}},$$

where 〈ROD〉 is the average ROD over a specific time interval and ROD is given by Eq. (3).

## 3 Results

### 3.1 Ionospheric irregularities during the 27th of August, 2015 geomagnetic storm

**Figure 2:**
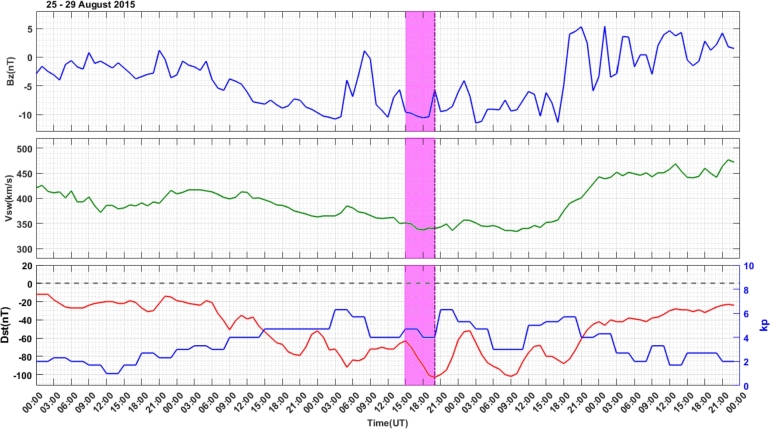
Variations in the IMF-Bz (top Panel), Vsw (middle Panel), and Dst and kp indices (bottom Panel) in the storm event of August 25 – 29, 2015. The storm main phase occurs during at 15:00 UT – 20:00 UT is indicated by the pink color bar.

The geomagnetic storm activity during August 25 – 29, 2015, is presented in Fig. 2. This figure illustrates solar wind speed, Vsw (km/s), interplanetary magnetic field component, IMF-Bz (nT), Dst index (nT), and kp index. On August 27, 2015, Earth experienced an intense G5 level geomagnetic storm, triggered by solar activity that include multiple plasma explosions and magnetic field disruptions. This powerful storm was characterized by several minimum values of the Dst index. For our study, we selected a minimum Dst index of -103 nT, which occurred at 20:00 UT during the nighttime, accompanied by a kp index of 6.3. The storm's main phase occurred at 15:00 UT – 20:00 UT, as indicated by the pink vertical color bar. During the main phase of the storm, IMF-Bz varies between -5.7 nT and -10.4 nT in the southward direction. Vsw attains a value between 362 km/s and 341 km/s; Dst index is between -72 nT and -103 nT, whereas the kp index start at a value of 4.7 and then decreases to 4 finally it attains a value of 6.3. In the recovery phase of the storm, the value of IMF-Bz varied between -11.5 nT and -4.1 nT until August 28, 2015, at 17:00 UT, and then increased to a value of 5.3 nT and turned northward. Vsw attained almost a constant value of 357 km/s at 17:00 UT and then increased to 469 km/s, whereas Dst started to increase at 18:00 UT on August 28, 2015.

**Figure 3:**
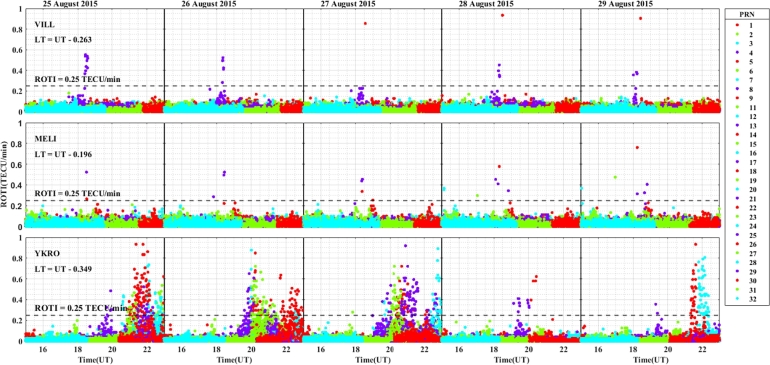
Daily variation of Rate of change TEC index (ROTI) for each PRN at stations VILL, MELI and YKRO during the storm of August 25 — 29, 2015 in the longitudinal sector of 15^∘^W – 0^∘^.

**Figure 4:**
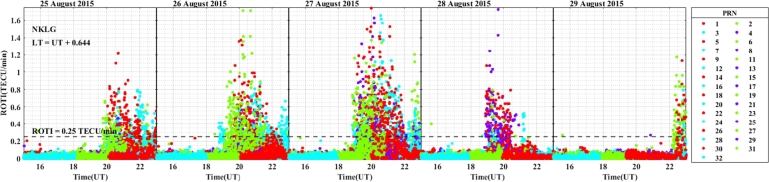
Daily variation of Rate of change TEC index (ROTI) for each PRN at stations VILL, MELI and YKRO during the storm of August 25 — 29, 2015 in the longitudinal sector of 0^∘^ – 15^∘^E.

**Figure 5:**
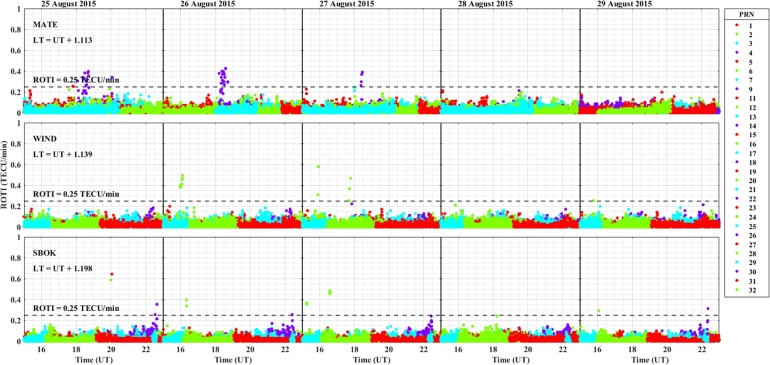
Daily variation of rate of TEC index indicated by ROTI for each PRN in the longitudinal sector of 15^∘^E – 30^∘^E (at stations MATE, WIND and SBOK) during 25 – 29 August, 2015 storm.

**Figure 6:**
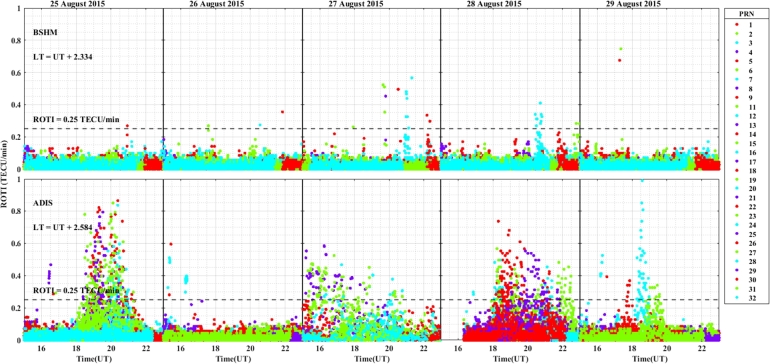
Variations rate of TEC index indicated by ROTI for each PRN in the longitudinal sector of 30^∘^E – 45^∘^E (at stations BSHM and ADIS) during 25 – 29 August, 2015 storm.

The daily variation of ROTI in the longitudinal sectors of 15^∘^W – 0^∘^, 0^∘^ – 15^∘^E, 15^∘^E – 30^∘^E and 30^∘^E – 45^∘^E during the 25 – 29 August 2015 storm event is displayed in Figure 3, Figure 4, Figure 5, Figure 6. We can observe that there is no occurrence of irregularity in the middle latitude region (at stations VILL and MELI) in the main and recovery phases of the storm since the value of ROTI < 0.25 TECU/min (see Fig. 3). Strong irregularity occurred at station NKLG during the main phase (August 27, 2015) and moderate irregularity in the recovery phase of the storm (28 and 29 August, 2015) as seen in Fig. 4. At station YKRO, a moderate irregularity was observed during the main phase (27 August, 2015) and recovery phase (29 August, 2015). In this case study at stations MATE, WIND and SBOK which are located between the eastern and western regions (in the middle of the study area, refer to Fig. 5) irregularity was not observed since in these stations the value of ROTI < 0.25 TECU/min. Finally, in Fig. 6, during the main and recovery phases of the storm, at station BSHM (located in mid-latitude), irregularity was not observed. At station ADIS (eastern equatorial region) moderate irregularities were observed on 27 August and 28 – 29 August, 2015 in the main and recovery phases of the storm respectively.

The value of ROTI is greater than 0.5 TECU/min in all longitudinal sectors of the equatorial region at stations ADIS (eastern region), YKRO (western region) and NKLG (eastern region). During the main phase of the geomagnetic storm period, ionospheric irregularity favors the low-latitude and equatorial region to occur, and the possible mechanism for the generation of these irregularities in this region is the gravitational RT-instability mechanism. This instability results from the interaction between the Earth's gravity and the charged particles (ions and electrons) in the ionosphere. When the F layer is lifted during sunset (18:00 – 22:00 LT), it leads to the formation irregularities, in which the strength of the observed irregularities dependent on different drivers. The interaction between the charged particles (ions and electrons) in the ionosphere with the Earth's gravity causes this instability, which is shown as irregularities when the F layer is lifted at sunset (between 16:00 – 22:00 LT).

During nighttime, the electric field in the ionosphere exhibits a westward direction, influenced by various factors, including solar radiation and geomagnetic conditions. The E×B drift results from the interaction between the electric field (E) and the Earth's magnetic field (B). When the electric field points westward (opposite to the Earth's rotation), it opposes the eastward plasma drift caused by the Earth's rotation. This E×B drift inhibits the vertical plasma motion (upward or downward), affecting the Total Electron Content (TEC). The decrease of TEC in the ionosphere occurs because the E×B drift counteracts the upward plasma motion. As a result, the ionospheric electron density decreases, leading to lower TEC values during nighttime. The duration and the strength of the irregularity occurrence in the western equatorial region is greater as compared to the eastern equatorial region. In the mid-latitude region the value of ROTI is less than 0.25 TECU/min, which indicates in all the stations located in mid-latitude region, for all the longitudinal sectors we considered in this case study, irregularities did not occur, and this is possibly due to the lack of RT-instability mechanism in the region.

### 3.2 Ionospheric irregularities during the 7th of October, 2015 geomagnetic storm

**Figure 7:**
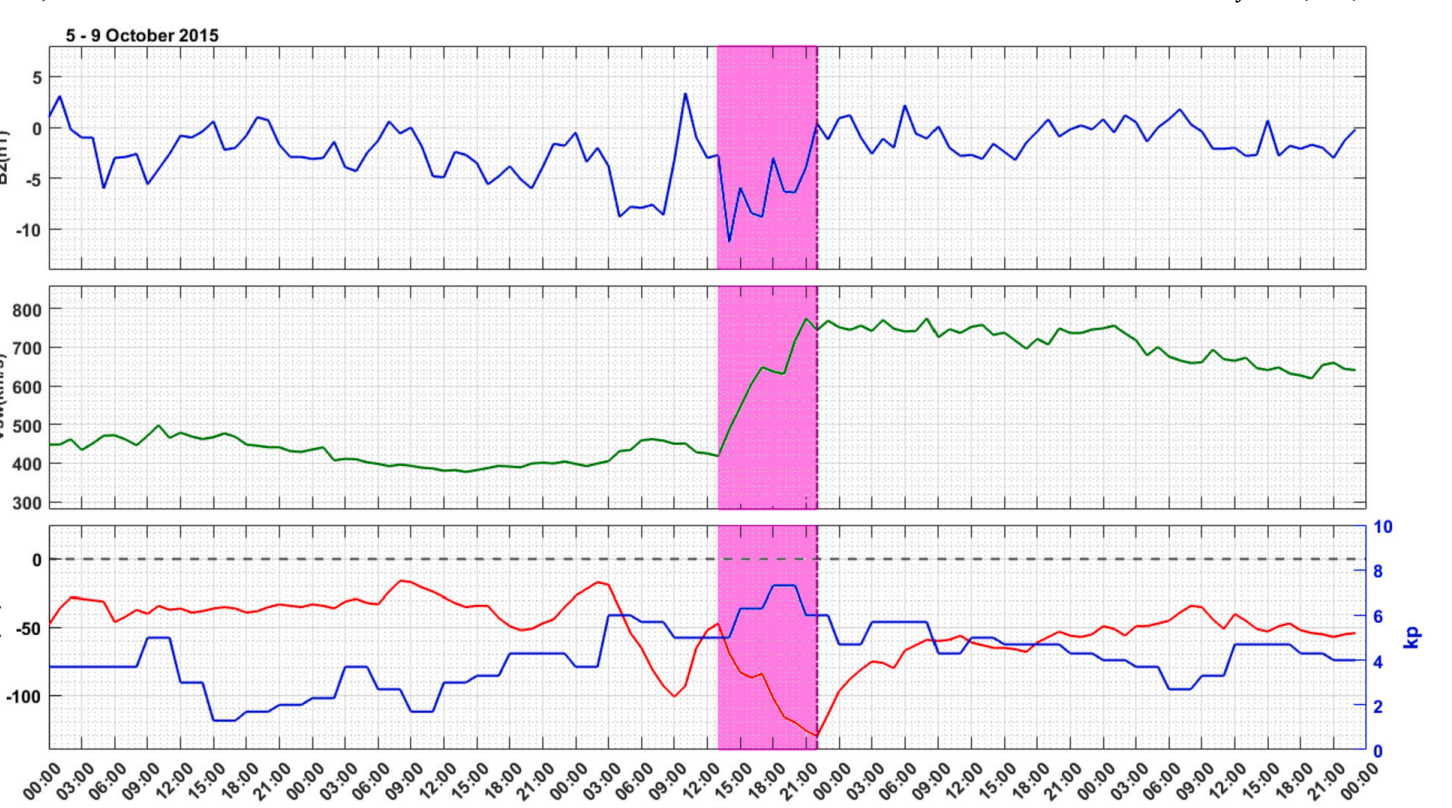
Variations in the IMF-Bz (top Panel), Vsw (middle Panel), and Dst and kp indices (bottom Panel) in the storm event of October 5 – 9, 2015. The storm attains minimum Dst-index value from 13:00 UT – 21:00 UT indicated by pink color bar.

The geomagnetic storm during 5 – 9 October, 2015 with variations of IMF-Bz, Vsw and Dst and kp indices are illustrated in Fig. 7 from the 1${}^{st}$ panel, 2${}^{nd}$ panel and 3${}^{rd}$ panel respectively. On October 7, 2015, Earth experienced a strong G3 level geomagnetic storm. The storm originated from the interaction with a High-Speed Stream (HSS) preceding a possible polarity coronal hole. During this event, there are two minimum values of Dst index, from these values, we took Dst index value fell below -120 nT and kp index reached 7. In this storm event, IMF-Bz changes from -2.7 nT – 11.3 nT in the southward direction and then oscillates between north and south direction with a value between -11.3 nT and 0.4 nT between south and north at 14:30 UT and 21:30 UT of 7 October, 2015 respectively, and then it turns to the northward direction. Vsw starts to increase from 418 km/s to 775 km/s, the Dst index starts to decrease from -47 nT to - 130 nT and the kp index increases from 5 to 7.3 values. In the recovery phase of the storm, IMF-Bz varies between - 3.2 nT and 2.2 nT, Vsw attains almost a constant value of 745 km/s up to 9 October, 2015, at 01:00 UT and then starts to decease its value by approximately 600 km/s, Dst index starts to increase whereas kp index decreases.

**Figure 8:**
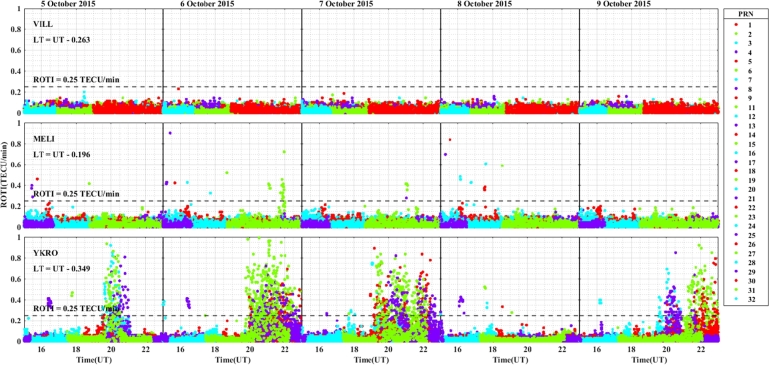
Daily variation of rate of TEC index indicated by ROTI for each PRN in the longitudinal sector of 15^∘^W – 0^∘^ (at stations VILL, MELI and YKRO) during 5 – 9 October, 2015 storm.

**Figure 9:**
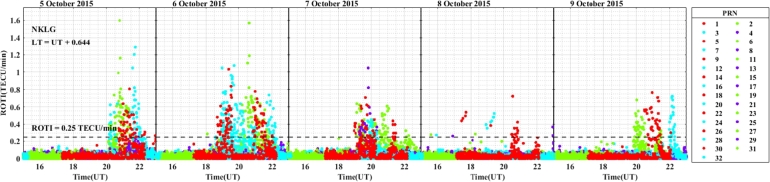
Daily variation of rate of TEC index indicated by ROTI for each PRN in the longitudinal sector of 0^∘^ – 15^∘^E (at station NKLG) during 5 – 9 October, 2015 storm.

**Figure 10:**
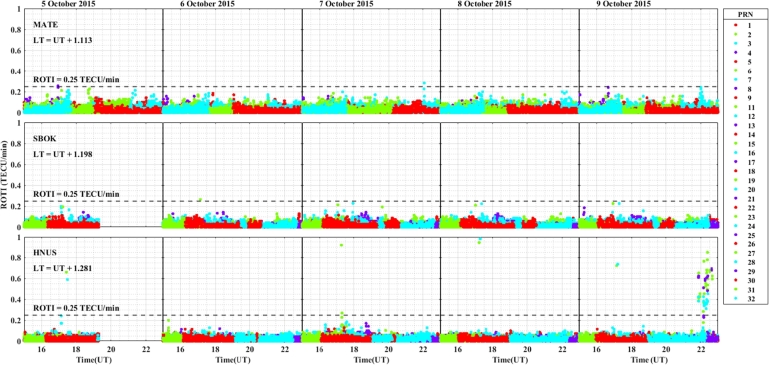
Daily variation of rate of TEC index indicated by ROTI for each PRN in the longitudinal sector of 15^∘^E – 30^∘^E (at stations MATE, SBOK and HNUS) during 5 – 9 October, 2015 storm.

**Figure 11:**
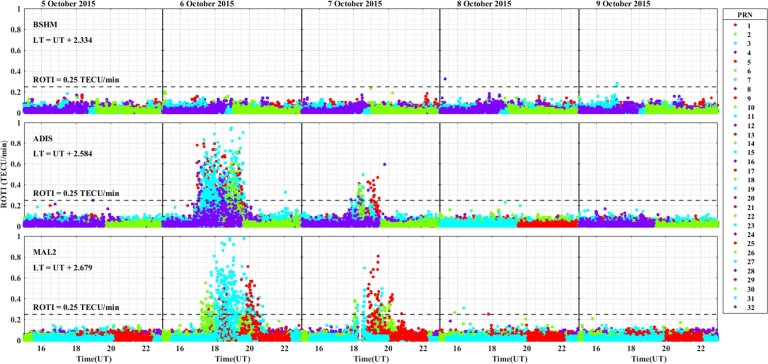
Variations rate of TEC index indicated by ROTI for each PRN in the longitudinal sector of 30^∘^E – 45^∘^E (at stations BSHM, ADIS and MAL2) during 5 – 9 October, 2015 storm.

In the longitudinal sector of 15^∘^W – 0^∘^, 0^∘^ – 15^∘^E, 15^∘^E – 30^∘^E and 30^∘^E – 45^∘^E during the intense geomagnetic storm event of 5 – 9 October, 2015, the daily variations of ROTI were depicted in Figure 8, Figure 9, Figure 10, Figure 11. In the main and recovery phases of the storm, as seen in Fig. 8, there is no irregularity occurrence at stations VILL and MELI. At station YKRO, irregularities were observed during the main and recovery phases of the storm. In the main phase of the storm, we observed strong irregularities on 7 October, 2015. In the recovery phase, moderate irregularity occurred on 9 October, 2015 but no irregularity on 8 October, 2015. In the main and recovery phases of the storm, in Fig. 9 at station NKLG, we observed irregularities. On 7 October 2015, during the main phase of the storm, moderate irregularities occurred. At stations MATE, SBOK and HNUS, the variation of ROTI was represented in Fig. 10, here there is no irregularity in the main and recovery phases of the storm which is similar to the result obtained during the August storm. The irregularities at stations BSHM, ADIS and MAL2 based on the value of were displayed in Fig. 11. There is no irregularity at station BSHM in the main and recovery phases of the storm. At stations ADIS and MAL2 the value of ROTI > 0.25, we observed moderate irregularity during the main phase of the storm (7 October, 2015), weak and moderate irregularity was observed in these stations whereas in the recovery phase of the storm irregularities were inhibited.

For the October storm, comparing the three longitudinal sectors, we observe irregularity: in the longitudinal sector of 15^∘^W – 0^∘^ and 0^∘^ – 15^∘^E of the western and eastern equatorial regions (at stations YKRO and NKLG respectively) in the main phase of the storm and 30^∘^E – 45^∘^E in the eastern equatorial region at stations ADIS and MAL2 during the main phase of the storm. The reason for these irregularities may be the vertical E×B drift refers to the motion of plasma along the magnetic field lines (E×B drift). During nighttime, the electric field (E) can be west ward. This westward E-field drives upward vertical E×B drifts in the ionosphere. These drifts transport plasma upward, affecting electron density distribution. When the vertical E×B drifts are enhanced, the growth of ionospheric irregularities occurred through RT-instability mechanism. The strength and duration of the observed irregularities were large in the western as compared to the eastern region

### 3.3 Ionospheric irregularities during the 20th of December, 2015 geomagnetic storm

**Figure 12:**
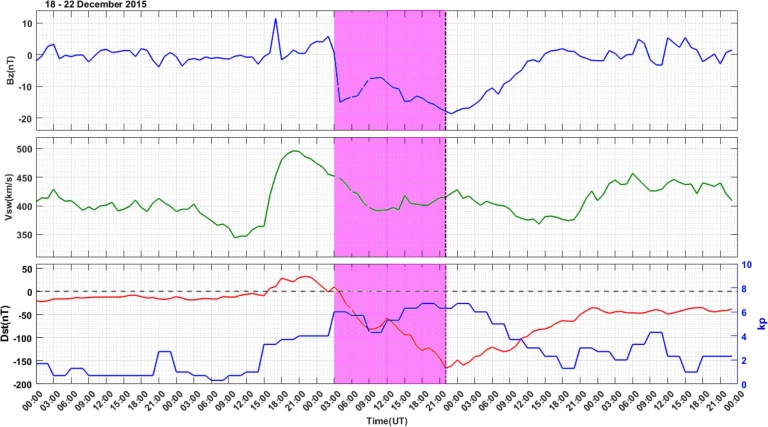
Variations in the IMF-Bz (top Panel), Vsw (middle Panel), and Dst and kp indices (bottom Panel) in the storm event of December 18 – 22, 2015. The storm attains minimum Dst-index value from 04:16 UT – 21:00 UT indicated by pink color bar.

On December 20, 2015, the Earth experienced a moderate G2 geomagnetic storm. This event was triggered by an enhanced solar wind environment and an extended period of southward magnetic field associated with the passage of a coronal mass ejection. The variations of the solar wind and geomagnetic parameters during the study period of 18 – 22 December, 2015 were depicted in Fig. 12. The IMF-Bz, Vsw, Dst & kp indices were represented from the top to bottom panels. During the main phase of the storm, IMF-Bz varies between -8.8 nT and -17.9 nT in the southward direction, Vsw varies in the range 393 km/s to 417 km/s, Dst index decreases from -57 nT to - 166 nT and kp index increases from 5.2 to 6.7. In the recovery phase of the storm, on 21 December, 2015, IMF-Bz start to increase in the northward direction and attained a value of 1.9 nT at 18:00 UT and then oscillate between 4.9 nT and -3.3 nT in the north and south directions. Vsw decreases to a value of 376 km/s at 20:00 UT and started to grow to attain a value of 457 km/s. The Dst index increases and attains almost a constant value of -36 nT at 24:00 UT and kp index decreases to a value of 1.3 at 18:00 UT.

**Figure 13:**
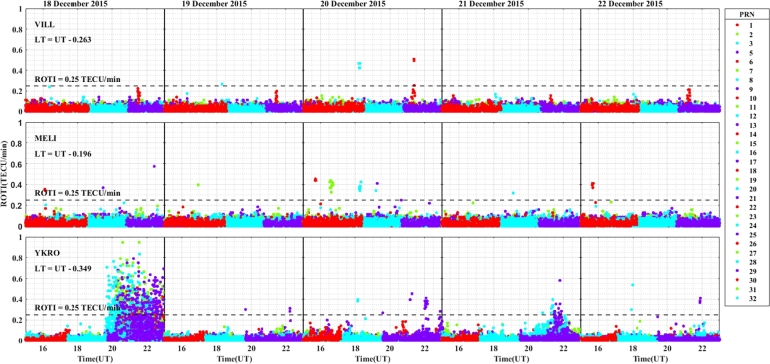
Daily variation of rate of TEC index indicated by ROTI for each PRN in the longitudinal sector of 15^∘^W – 0^∘^ (at stations VILL, MELI and YKRO) during 18 – 22 December, 2015 storm.

**Figure 14:**
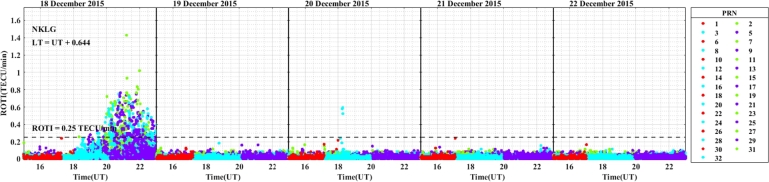
Daily variation of rate of TEC index indicated by ROTI for each PRN in the longitudinal sector of 0^∘^ – 15^∘^E (at station NKLG) during 18 – 22 December, 2015 storm.

**Figure 15:**
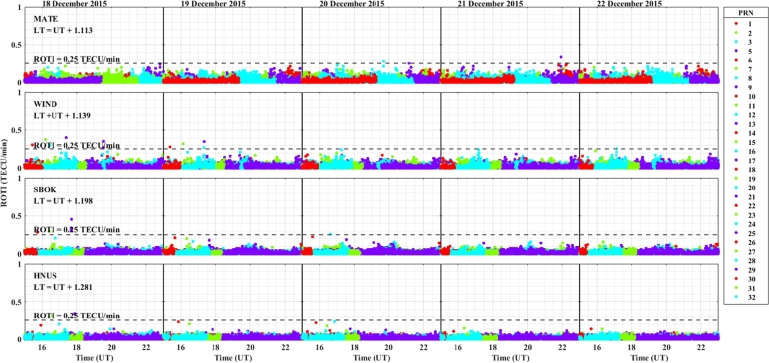
Daily variation of rate of TEC index indicated by ROTI for each PRN in the longitudinal sector of 15^∘^E – 30^∘^E (at stations MATE, WIND, SBOK and HNUS) during 18 – 22 December, 2015 storm.

**Figure 16:**
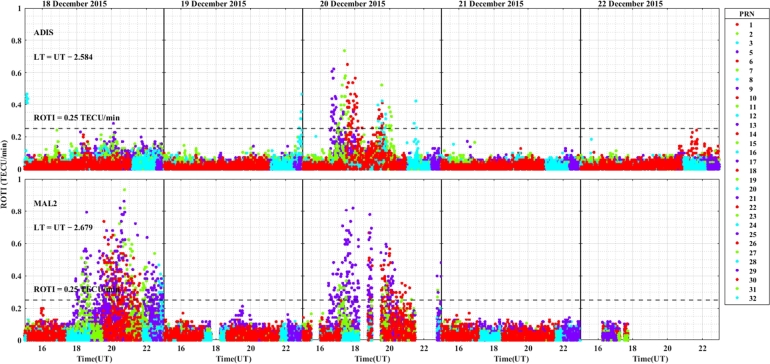
Variations of rate of TEC index indicated by ROTI for each PRN in the longitudinal sector of 30^∘^E – 45^∘^E (at stations ADIS and MAL2) during 18 – 22 December, 2015 storm.

For the geomagnetic storm event that occurred on 18 – 22 December, 2015 in the longitudinal sectors of 15^∘^W – 0^∘^, 0^∘^ – 15^∘^E, 15^∘^E – 30^∘^E and 30^∘^E – 45^∘^E the observed irregularity using the variation of ROTI was illustrated in Figure 13, Figure 14, Figure 15, Figure 16. We observed that no irregularity at stations VILL and MELI in both the main and recovery phases of the storm as displayed in Fig. 13. At station YKRO in Fig. 13, we observed weak irregularity in the recovery phase on 21 December, 2015. There is no irregularity observed in the main phase of the storm in this station. As seen in Fig. 14, irregularities were not observed in the main and recovery phases of the storm at the station NKLG. For stations MATE, WIND, SBOK and HNUS as shown in Fig. 15 we cannot observe irregularity in the main and recovery phases of the storm, this result is similar to the results obtained in the August and October storm events. At station ADIS we observed a moderate irregularity in the main phase of the storm on 20 December, 2015. In the recovery phases of the storm, irregularity were inhibited in this station. At station MAL2, a moderate irregularity occurred in the main phase (20 December, 2015) and we cannot observe irregularity in the recovery phase of the storm. At this station, the gap on 22 December, 2015, represents there is no observational data as illustrated in Fig. 16. The possible mechanism for the generation of irregularities in low and equatorial latitudes, is the same as described in the prior storm events. For the absence of irregularities in mid-latitude is due to the lack of RT-instability in the region.

The strength of the observed irregularities using the values of ROTI in all the stations used in this case study, in each of the longitudinal sectors considered, during the main phases of the geomagnetic storm events of 27 August, 7 October and 20 December, 2015 with the respective occurrence time in UT were summarized in Table 3.

**Table 3:** Maximum ROTI (in TECU/min) with the corresponding occurrence of time (in UT) during 27 August, 7 October, and 20 December 2015 storm events.

Long. Sec	Storm Day	Station	ROTI	Time	Classification
		VILL	0.22	18:00	No irregularity
	August 27	MELI	0.19	19:00	No irregularity
		YKRO	0.72	21:00	Moderate irregularity
		VILL	0.12	18:00	No irregularity
15^∘^W – 0^∘^	October 7	MELI	0.23	21:00	No irregularity
		YKRO	1.41	19:30	Strong irregularity
		VILL	0.11	20:00	No irregularity
	December 20	MELI	0.24	20:00	No irregularity
		YKRO	0.36	20:00	Weak irregularity

	August 27	NKLG	1.65	20:00	Strong irregularity
0^∘^ – 15^∘^E	October 7	NKLG	0.82	19:00	Moderate irregularity
	December 20	NKLG	0.18	18:00	No irregularity

		MATE	0.15	20:00	No irregularity
	August 27	WIND	0.14	18:00	No irregularity
		SBOK	0.15	20:00	No irregularity
		MATE	0.16	19:00	No irregularity
15^∘^E – 30^∘^E	October 7	SBOK	0.13	20:00	No irregularity
		HNUS	0.16	19:00	No irregularity
		MATE	0.21	20:00	No irregularity
		WIND	0.24	17:00	No irregularity
	December 20	SBOK	0.18	17:00	No irregularity
		HNUS	0.13	17:00	No irregularity

	August 27	BSHM	0.46	21:00	Weak irregularity
		ADIS	0.57	16:30	Moderate irregularity
		BSHM	0.23	18:00	No irregularity
30^∘^E – 45^∘^E	October 7	ADIS	0.46	19:30	Moderate irregularity
		MAL2	0.81	19:40	Moderate irregularity
	December 20	ADIS	0.73	17:40	Moderate irregularity
		MAL2	0.82	18:00	Moderate irregularity

## 4 Measurements of in situ electron density and calculated RODI values from SWARM satellites

**Figure 17:**
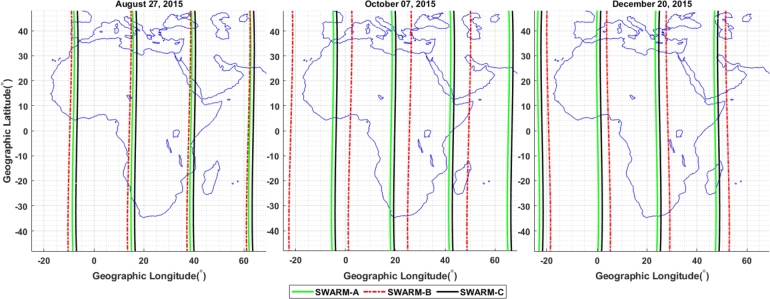
Trajectory for Swarm A, Swarm B and Swarm C is respectively represented by green, red, and black lines on August 27, October 7, and December 20, 2015.

We recorded the electron density data from the Swarm constellation of Swarm A, B, and C during the geomagnetic storm events of 25 – 29 August, 5 – 9 October, and 18 – 22 December, 2015, to highlight the presence of the top-side ionospheric irregularity at nighttime over the African region in different longitudinal sectors at various latitudes. The trajectories of Swarms A, B, and C crossing our case study area during the geomagnetic storm events of August 27, October 7, and December 20, 2015 are illustrated in Fig. 17. As their constellations imply, here we can observe that the paths of Swarm A and C are close to each other and Swarm B displayed at a distance hence Swarm A and C flew side by side at almost same the altitude, whereas Swarm B flew at a higher altitude. Here, we plotted this figure only to show the trajectories of the three satellites.

**Figure 18:**
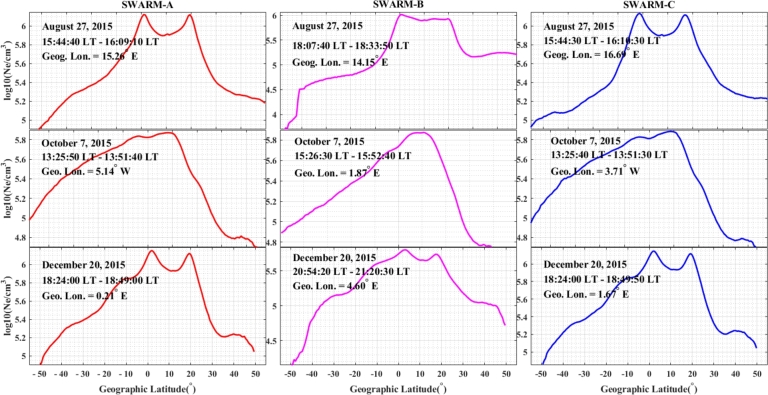
Variations in electron density with respect to geographic latitude for Swarms A, B, and C on storm days on August 27, October 7, and December 20, 2015, at a particular time.

In the storm events of 27 August, 7 October, and 20 December, 2015, the observed electron density in the nighttime at a particular time against latitude was presented in Fig. 18. In the specified storm periods in our case study area, for Swarm A, B, and C, large variations of Ne were detected in the Equatorial and low-latitude regions. The observed patterns for *Ne* in Swarm A and C were nearly identical since they flew alongside each other. The electron density variations for the three satellites occurred in the equatorial and low latitudes in the range of 10^∘^S – 20^∘^N during the storm events of August 27 (at a particular time of 15:44:40 – 16:09:10 LT and geographic latitude of ∼ 15.26^∘^ E for Swarm A, 18:07:40 – 18:33:50 LT and geographic latitude of ∼ 14.15^∘^ E for Swarm B, 15:44:30 – 16:10:30 LT and geographic latitude of ∼ 16.69^∘^ E for Swarm C), October 7 (at a particular time of 13:25:50 – 13:51:40 LT and geographic latitude of ∼ 5.14^∘^ W for Swarm A, 15:26:30 – 15:52:40 LT and geographic latitude of ∼ 1.87^∘^ E for Swarm B, 13:25:40 – 13:51:30 LT and ∼ 3.71^∘^ W for Swarm C) and December 20 (at a particular time of 18:24:00 – 18:50:00 LT and geographic latitude of ∼ 0.21^∘^ E for Swarm A, 20:54:20 – 21:20:30 LT and geographic latitude of ∼ 4.60^∘^ E for Swarm B, 18:24:00 – 18:49:50 LT and geographic latitude of ∼ 1.67^∘^ E for Swarm C).

We employed the RODI index to analyze the in situ electron density data from the Swarm satellites in order to examine the dynamics of top-side ionospheric irregularities during storms. The observed RODI value variations as a function of geographic latitude from the three Swarm satellites (A, B and C) during the August, October and December storm events were presented and explained as follows.

**Figure 19:**
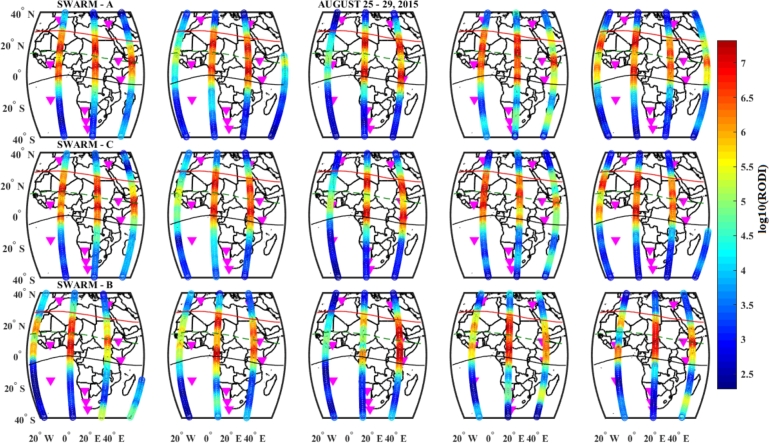
Variations of RODI values indicating top-side ionospheric irregularity observed by Swarm A (1^*st*^ panel), Swarm C (2^*nd*^ panel) and Swarm B (3^*rd*^ panel) on August 25 – 29, 2015 storm.

On 25 – 29 August, 2015, geomagnetic storm, the RODI distribution for the Swarm satellites (A, B, and C) presented in Fig. 19. The equatorial and low latitude regions within $15^{\circ }$W and $15^{\circ }$E, have substantially higher RODI values, as a result of this, small scale top-side ionospheric irregularities were observed in these regions. On the other hand, in the northern and southern regions outside of the geomagnetic equator, the values of the observed RODI are small due to this, top-side ionospheric irregularities were not observed. The RODI distributions for Swarm A and C are remarkably comparable, and we can also see that there was significant top-side irregularity during the storm's early and late phases. Comparing Swarm B to Swarm A and C, the value of the RODI is larger in Swarm A and C than in Swarm B, this implies that the irregularity observed by Swarm A and C was stronger than Swarm B in the main phase of the storm.

**Figure 20:**
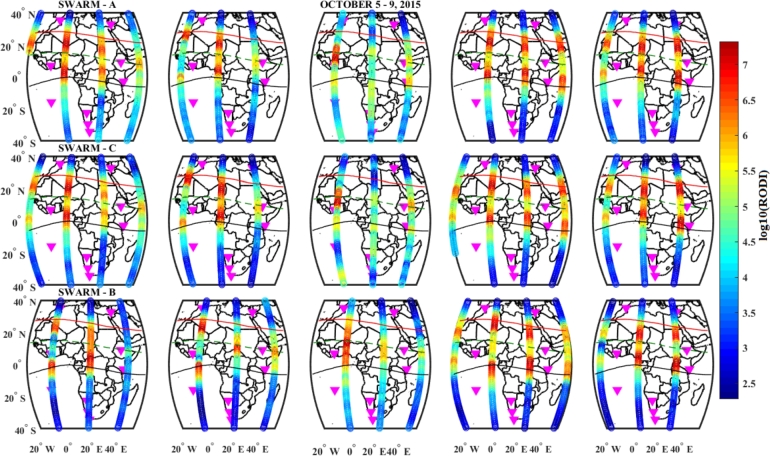
Variations of RODI values indicating top-side ionospheric irregularity observed by Swarm A (1^*st*^ panel), Swarm C (2^*nd*^ panel) and Swarm B (3^*rd*^ panel) on October 5 – 9, 2015 storm.

For Swarm A, B, and C (first, second, and third panels), the distribution of RODI during the geomagnetic storm event of 5 – 9 October, 2015, was illustrated in Fig. 20. The equatorial and low latitude regions were exhibited substantial large RODI values in which strong top-side ionospheric irregularities were observed in these regions. The RODI values obtained from the in situ electron density using Swarm A and C are almost similar. The calculated value of RODI in Swarm B is higher than in Swarm A and C. In the main phase of the storm, the irregularity observed by Swarm B was stronger than Swarm A and C. The observed irregularity pattern for Swarm A and C are almost similar, since these two satellites flew side by side. Outside the equatorial region in the northern and southern regions in our case study, irregularity was not observed.

**Figure 21:**
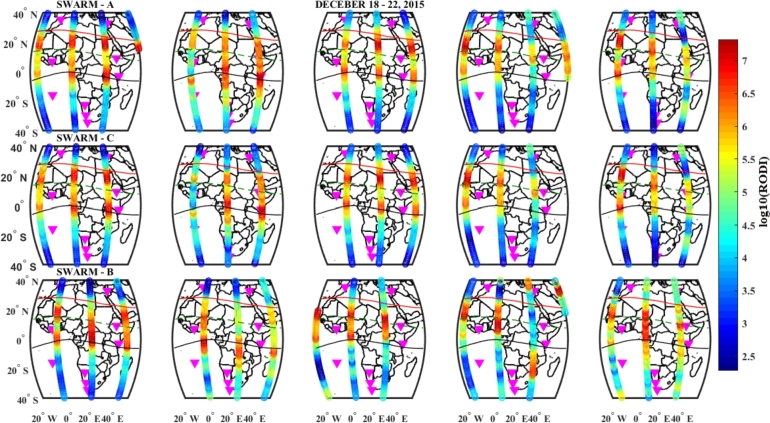
Variations of RODI values indicating top-side ionospheric irregularity observed by Swarm A (1^*st*^ panel), Swarm C (2^*nd*^ panel) and Swarm B (3^*rd*^ panel) on December 18 – 22, 2015 storm.

The RODI value distribution pattern for the three Swarm satellites observed during 18 – 22 December, 2015, storm event was presented in Fig. 21. In the main phase of the storm, the observed RODI values were greater for Swarm B as compared to Swarm A and C and hence strong irregularity were observed by Swarm B. In this storm event, strong irregularity was observed in the low and equatorial regions.

The RODI values on December 20 were higher than those on August 27 and October 7, and we can also see that the RODI value on August 27 is higher than that on October 7, during the main phases of the three storm events, as shown by the three Figures (19, 20 and 21). This suggests that during December, there was a significant intensity of top-side irregularities observed. The region between 15^∘^S and 15^∘^N had the highest RODI values across all storm occurrences we looked at for this case study. As a result, we saw increased top-side irregularities in the low-latitude and equatorial regions. Therefore, the observed top-side ionospheric irregularities for the three Swarm satellites were strong during the December storm event as compared to the October and August storm events.

In the vicinity of the geomagnetic equator, ionospheric irregularities in the top-side ionosphere occur after sunset and sometimes past midnight. They manifest as structures that are field-aligned and exhibit reductions in plasma density. The unstable Rayleigh-Taylor process, which causes ionospheric plasma bubbles to expand when the electron density differential in the ionosphere's lower layers rises, is the underlying mechanism of these irregularities. These bubbles expand due to RT-instability, upsetting the ionospheric equilibrium.

In this study, we observed that, maximum ROTI values were obtained in the low and equatorial regions using ground-based observations and RODI values from the LEO satellites were maximum in the region between 15^∘^S to 15^∘^N. From this, we can say that, the results obtained from the in-situ measurements by the Swarm satellites and ground-based measurements using GPS satellites, show similar characteristics interims of occurrence of time and spatial distribution. Ionospheric irregularities observed from ground-based measurements occurred in low and equatorial regions while top-side ionospheric irregularities were associated with the magnetic equator regions. For both ground-base and LEO satellite observations, the irregularities occurred during the post-sun set hours, and irregularities were not observed in mid-latitudes during our study periods.

## 5 Discussions

In general, irregularities in the ionosphere are often linked to an increase in the F-layer due to the influence of PRE in the E×B drift. This rise in altitude can create favorable conditions for the occurrence of irregularities through the RT-instability [45], [46]. Several indices have been used to report the influence of geomagnetic storms regarding the incidence of ionospheric irregularities. For instance, Aarons [47], Aarons et al. [33] and Tulasi Ram et al. [48] claimed that when storm-driving mechanisms increase (or decrease) the E×B vertical drift that causes the F-region to be lifted upward to higher altitudes, irregularities may be initiated (or inhibited) during a storm.

The present study investigates the longitudinal variation of nighttime ionospheric irregularities at various latitudes during the geomagnetic storm events of August 27, October 7, and December 20, 2015, over the Europe-African longitudinal sectors. The observed irregularity pattern presented in this paper related to the storm timing at each station. This could be related to the equatorial vertical drift, which is known to change significantly with longitude [49].

ROTI variation during the August storm event for different longitudinal sectors were presented in Figure 3, Figure 4, Figure 5, Figure 6. Ionospheric irregularities occur more frequently during disturbed geomagnetic conditions [50], which show considerable longitudinal disparities. Ionospheric irregularities over the African sector show a wide range of variations, in strength and latitudinal variations within the same longitudinal sector [51]. During the main phases of the storm, irregularities were noted in the equatorial latitude at YKRO (long. ∼ 5.240^∘^W), NKLG (long. ∼ 9.672^∘^E) and ADIS (long. ∼ 38.766^∘^E) [50], [52]. Since in low and equatorial regions, during the evening, when open circuit conditions are met, the winds the polarized electric field. Hence, the positive and negative charges create an electric field (E) that interacts with the horizontal magnetic field (B) in the vicinity of the geomagnetic dip equator. The ionospheric plasma is propelled by the resulting drift velocity [11]. The E×B drift near the equator undergoes a significant increase and changes its polarity near the sunset terminator, which is known as pre-reversal enhancement (PRE) [53], [54]. It has been observed that during nighttime, the plasma density in the E region becomes diluted due to chemical recombination, resulting in a loss of its ability to counteract the electric field generated by the dynamo in the F-region. The combination of the rapid rise of the F-layer and the steep vertical density gradient at the equatorial region creates the conditions for the RT-instability in the nighttime ionosphere, making it susceptible to the occurrence of plasma irregularities. Nighttime irregularities were absent in mid-latitude at stations VILL (long. ∼ 3.952^∘^W), MELI (long. ∼ 2.952^∘^W), MATE (long. ∼ 16.704^∘^E), WIND (long. ∼ 17.089^∘^E), SBOK (long. ∼ 17.979^∘^W) and BSHM (long. ∼ 35.023^∘^E) [51], [52]. At mid-latitudes, irregularities are rarely occurring, except throughout the solar cycle's most active years, when there are strong ionospheric storms. Hence, in our case study, all the geomagnetic storms considered are not extreme events, hence no irregularity occurrence at these regions [52]. This due to the lack of RT-instability and the absence of direct connection to the strong forcing from the magnetosphere [14], [15]. All these results indicate that the observed nighttime irregularities vary in latitude within the Europe-African longitudinal sector.

We observed the enhancement of irregularity at stations YKRO, NKLG, ADIS and MAL2 in the main phases of the storm (see Figure 3, Figure 4, Figure 6, Figure 8, Figure 9, Figure 11 an 16). Hence, all these results indicate that the observed irregularity attains significant longitudinal variations in our study area. The results presented in these figures, show notable longitudinal variations in the incidence of ionospheric irregularities during the main phase of the geomagnetic storm. The observed ionospheric irregularities indicate a wide range of fluctuations in the African sector (at stations ADIS and MAL2). These variations include enhancement and suppression of the irregularity's strength. During the main phase, nighttime irregularities were noted at YKRO, NKLG, ADIS and MAL2 in the equatorial region [52], [50] and are absent at stations VILL, MELI, MATE, WIND, SBOK, and BSHM. This indicates that the observed irregularities exhibit significant longitudinal variation within our study area. All the above results obtained in our case study are similar to the results obtained by [27], [55], [15].

In the main phase of geomagnetic storms, the presence or disappearance of low latitude and equatorial region ionospheric irregularity can vary significantly. Geomagnetic storms often enhance ionospheric irregularities, leading to increased scintillation. This is particularly true at low-latitude and equatorial regions where the ionosphere is more susceptible to disturbances [56], [57]. During the storm main phase, electric fields from the magnetosphere can penetrate to low latitudes, causing rapid changes in ionospheric conditions. These fields can enhance the E×B drift, which results in the formation of plasma motion, which are a major cause of electron density variation, results ionospheric irregularity. The occurrence of irregularity can be highly variable during storms. While some regions may experience intense irregularity, others might see a temporary inhibited irregularity due to variations in the ionospheric currents and electric fields [47], [33], [48].

In this study, when compared to the observations made in the mid-latitude with that of equatorial and low-latitude, the situation in equatorial and low-latitude is very different in the mid-latitude region, due to in low/equatorial regions irregularities were generated due to gravitational RT-instability [9], [10], [11] but not in mid-latitude [14], [15]. The Prompt Penetration of Electric Field (PPEF), which is injected into the low-latitude during the main phase of the storm, occurred around the post-midnight hour, a time that may have favored the occurrence of irregularities in the equatorial and low-latitude whereas in the mid-latitude results in suppression of irregularities. The equatorial region's ionosphere is particularly susceptible to changes in electric fields because of the region's distinct magnetic orientation. Strong electric fields that emerge from the magnetosphere can reach low latitudes during geomagnetic storms [58]. The injection of the electric field moving westward during the main phase could have encouraged the emergence of irregularities and accelerated the typical upward plasma drift.

Additionally, the suppression of irregularities could be a sign of the influence of additional storm-induced related drivers, whose actions could result in a mechanism that might not be favorable to the upward motion of plasma [59], [60], [61]. Such drivers may include the action of a westward (i) PPEF of magnetospheric origin due to the northward orientation of IMF-Bz and (ii) disturbance of the dynamo electric field (DDEF) due to storm-induced equatorward wind (e.g., [62], [59], [60]).

The top-side ionospheric irregularities which are seen in the post-sunset sector close to the geomagnetic equator, are caused by field-aligned structures that move upward and are characterized by plasma density depletions, or what are commonly called EPBs [63]. The presence of F region plasma irregularities in the low-latitude/equatorial region has been linked to an unstable RT-instability mechanism during the post-sunset period [11], [64], [65]. These EPIs begin at the F region on the bottom side, and polarization electric fields at night are crucial to their non-linear ascent from the bottom side and appearance at the top side.

The top-side in situ electron density variations were illustrated in Fig. 15. We observe that the Equatorial and low-latitude regions experienced significant differences in electron density, which led to significant variations in the RODI values found in these locations across the African sector [66], [43], [28]. We see more plasma density fluctuations in the equatorial and low-latitude regions as compared to the northern and southern hemispheres for this study. Very irregular ionospheric plasma density in low-latitude and equatorial regions is visible, and this is connected to both equatorial plasma bubbles and post-evening Equatorial Spread F in the study area [67]. However, as shown in Figure 16, Figure 17, Figure 18, substantial large variations of RODI values were observed in the low and equatorial regions, suggesting that strong top-side ionospheric irregularity was observed. By referring to our study area, the distribution of RODI was found to be stronger (more intense) in the low-latitude and equatorial regions as compared to the northern and southern sectors [67], [66], [43], [28], [29].

## 6 Conclusions

In the present study, we examined the characteristics of nighttime ionospheric irregularities at various longitudinal sectors during geomagnetic storm events of August 27, October 7, and December 20, 2015. Enhanced irregularities are only observed during these disturbed geomagnetic conditions in the equatorial and low-latitude regions over the African sector, specifically in the main phase at nighttime. However, the intensity of the irregularities that were observed decreases as we moved eastward, likely due to the presences of an eastward directed electric field at nighttime.

The occurrence of irregularities differed in strength from one sector to another. During the main phases of the storm events, irregularities have been noted at various longitudinal sectors (at stations YKRO, NKLG, ADIS, and MAL2 which are situated in the north-west, north-east, north-east, and south-east, respectively) across the African region. These stations are all situated within the latitudinal range between $15^{\circ }$S and $15^{\circ }$N.

In this study, we found that geomagnetic storm activity significantly impacted the existence of nighttime irregularities in the ionospheric layer in the low latitude and equatorial regions over several longitudinal sectors over the Africa. When the Dst index attains its maximum trough value, nighttime ionospheric irregularities are noticed, which vary with latitude and display distinct patterns in different longitudinal sectors.

In low-latitude and equatorial regions within the latitudinal range of $15^{\circ }$S and $15^{\circ }$N, the observed in situ electron density variation indicates enhanced top-side ionospheric irregularities. Along with the ground-based irregularities, these top-side irregularities became stronger during nighttime geomagnetic storm occurrences in these places.

## CRediT authorship contribution statement

**Yibekal Kassa:** Writing – review & editing, Writing – original draft, Validation, Software, Resources, Methodology, Investigation, Formal analysis, Data curation, Conceptualization. **Ambelu Tebabal:** Writing – review & editing, Visualization, Validation, Conceptualization. **Baylie Damtie:** Writing – review & editing, Visualization, Validation, Methodology.

## Declaration of Competing Interest

The authors declare that they have no known competing financial interests or personal relationships that could have appeared to influence the work reported in this paper.

## Data Availability

The authors wish to express their appreciation to the following organizations: the University of NAVSTAR consortium (UNAVCO) for the GPS data (https://www.unavco.org/data/gps-gnss/data-access-methods/data-access-methods.html), the ACE SWEPAM teams for providing the ACE data (https://omniweb.gsfc.nasa.gov/form/dx1.html) and Explore the European Space Agency's Earth Observation satellite for the Swarm data (https://vires.services/).
